# Predictors for Unfavorable Early Outcomes in Elective Total Hip Arthroplasty: Does Extreme Body Mass Index Matter?

**DOI:** 10.1155/2019/4370382

**Published:** 2019-10-07

**Authors:** Chun-Yu Hung, Chih-Hsiang Chang, Yu-Chih Lin, Shen-Hsun Lee, Szu-Yuan Chen, Pang-Hsin Hsieh

**Affiliations:** ^1^Department of Orthopaedic Surgery, Chang Gung Memorial Hospital, Linkou, Taiwan; ^2^Chang Gung University, Taoyuan, Taiwan

## Abstract

**Background:**

Studies of previous cohorts have demonstrated a controversial association between extreme body mass index (BMI) and complication rates following total hip arthroplasty (THA). The purpose of this study was to compare 30-day perioperative complications in underweight (BMI <18.50 kg/m^2^), normal-weight (BMI 18.50–24.99 kg/m^2^), overweight (BMI 25.00–29.99 kg/m^2^), class I obesity (BMI 30.00–34.99 kg/m^2^), and morbidly obese (BMI ≥35.00 kg/m^2^) groups.

**Methods:**

We performed a cohort study including patients who underwent unilateral primary THA by a single surgeon between January 2010 and December 2015 at our institution. We assessed 30-day complications, operation time, operative blood loss, and length of hospital stay.

**Results:**

We identified 1565 primary THAs that were performed in patients with varying BMI levels. Compared with the normal-weight patients, the morbidly obese group had a higher 30-day complication rate (8.9% vs. 2.4%), longer operative time (79 minutes vs. 70 minutes), and more blood loss (376 mL vs. 302 mL). Underweight patients did not present any 30-day complications, and there were no differences among underweight and normal-weight patients regarding complication rates, operative time, or blood loss. The mean length of hospital stay was comparable among the different BMI groups. In the multivariate regression model, higher BMI was not associated with a higher risk of 30-day complications. Independent risk factors for 30-day complications were advanced age, prolonged operative time, and cardiovascular comorbidities.

**Conclusion:**

Although increased operative time, blood loss, and perioperative complications were seen in the morbidly obese patients, BMI alone was not an independent risk factor for a higher 30-day complication rate. Therefore, our data suggest clinicians should make elderly patients aware of increased 30-day complications before the procedure, particularly those with cardiovascular comorbidities. Withholding THA solely on the basis of BMI is not justified.

## 1. Introduction

Total hip arthroplasty (THA) reduces pain and improves function and quality of life in the great majority of patients with a severely damaged arthritic hip [1–3]. In recent decades, the demand for THA has increased worldwide, accounting for a large share of the national health expenditure [4, 5]. In Taiwan, the number of THAs performed is projected to increase by 69.7% in 2030 compared to 2005 [6].

Obesity continues to be a troublesome public health issue because of the established health risks and substantial increase in prevalence [7]. A positive association between obesity and hip osteoarthritis has been studied intensively [8–11]. Rising levels of obesity coupled with projected growing demand for THA present a challenging problem for orthopedic surgeons [12]. Some studies have shown that obese patients are associated with a higher rate of perioperative complications following THA, including dislocations, deep wound infections, and venous thromboembolic disease [13–15]. Furthermore, obese patients are associated with longer hospital stays and higher costs than the general population [16, 17]. Conversely, other studies have not found any differences with regard to the above complications [18–20].

Apart from obese patients, underweight patients undergoing THA are also an issue within the arthroplasty community; however, they have been largely overlooked and understudied. Compared with normal-weight patients, underweight patients may incur a higher rate of postoperative dislocation, infection, and cardiac complications [21, 22]. However, a recent cohort study presents contradictory results and reports that there are no increased rates of infectious or medical complications in underweight patients undergoing THA [23].

With this conflicting background, we undertook a large, retrospective study in a high-volume referral center to examine whether the clinical outcomes of patients with extreme BMI levels differed from those of normal-weight patients.

## 2. Patients and Methods

### 2.1. Study Design

We retrospectively studied prospectively gathered data, analyzing the relationships between patient BMI and perioperative complications in primary THAs. The study protocol was approved by the Institutional Review Board of the Chang Gung Memorial Hospital. All THAs took place at a single institution, and a single experienced surgeon who performed more than 300 primary THAs per year during the study period participated in all of the operations. Our center is a tertiary referral hospital located in north Taiwan, treating 600 new cases of primary THA per year.

Demographic data were collected including age, sex, body mass index (BMI), surgical approach, etiology of hip pathology, and comorbidities. We classified patients using the World Health Organization definitions for BMI, as underweight (BMI <18.50 kg/m^2^), healthy (BMI 18.50–24.99 kg/m^2^), overweight (BMI 25.00–29.99 kg/m^2^), class I obese (BMI 30.00–34.99 kg/m^2^), and morbidly obese (≥35.00 kg/m^2^) groups [24]. At our institution, it is our practice not to refuse THA because of obesity.

All complications that occurred during the hospital stay and within a 30-day postoperative period were identified. Complications were then divided into surgery-related and surgery-unrelated categories. Surgery-related complications included intraoperative periprosthetic fracture, nerve injury, superficial wound infection, deep wound infection, dislocation, and deep vein thrombosis. Surgery-unrelated complications included periprosthetic fractures resulting from postoperative fall, acute renal failure, stroke, and readmission to hospital for any reason other than surgery-related complications during the first 30 days after THA. Reasons for readmission and the relationship with the index THA were recorded, while we excluded planned readmissions, such as scheduled spine surgery or contralateral side THA.

### 2.2. Eligibility

From January 2010 to December 2015, the results of 1987 consecutive primary THAs (in 1790 patients) performed by the senior surgeon were examined. We excluded patients who had coagulation abnormalities, radiation-induced femoral head necrosis, bilateral THA on the same day, and THA for acute fracture of the hip. For patients who received bilateral sequential THA during the period, data were recorded only for the first procedure. Overall, 1565 unilateral primary THAs in 1565 patients were eligible for inclusion in the study.

### 2.3. Surgical Technique

All enrolled patients received general anesthesia and orotracheal intubation. All surgical procedures were performed by a single surgeon using the transgluteal surgical approach, modified from the direct lateral approach described by Hardinge [25], and were the same as the technique described by Frndak et al. [26]. The prosthesis used in all patients was the Trilogy Acetabular Cup and VerSys Fiber Metal Taper Stem (Zimmer, Warsaw, IN, USA). The fixation of the cementless THA components was achieved primarily by the press-fit technique. Augmented screw insertion for supplemental acetabular cup fixation was not routine but was used when necessary as judged by the treating surgeon.

### 2.4. Postoperative Care

The postoperative care and rehabilitation protocol were standardized for all patients and included venous thromboembolic prophylaxis and intravenous antibiotics. Postoperatively, patients were allowed to mobilize to full weight-bearing movements as tolerated, using a walker in the first 4 weeks or for as long as they thought was necessary. Antibiotic prophylaxis was given routinely for 1 day, while anticoagulant prophylaxis continued for up to 35 days after surgery in the absence of contraindications [27].

### 2.5. Outcome Assessment

The primary outcome measures were 30-day complication rate, including surgery-related and surgery-unrelated complications.

### 2.6. Data Analysis

All analyses were performed using the SPSS statistical software, version 22.0 (SPSS Inc, Chicago, IL, USA). For the normally distributed numerical outcome data, one-way analysis of variance (ANOVA) and Scheffe post hoc tests were used for between-group comparisons. The Fisher exact test and chi-squared test were used to compare categorical data among the groups. The multivariate regression model included patient demographics, comorbidity, and operative variables with a univariate comparison *p* value of <0.05. Cofounders such as age, gender, cardiovascular comorbidities, and operation time were included in the regression model to investigate the relationship between the outcomes and BMI. The significance level was set at *p* < 0.05 for all univariate and multivariate analyses.

## 3. Results

### 3.1. Patient Characteristics

In total, 1565 primary THAs were carried out in 1565 patients who met the inclusion criteria. All 1565 patients with 1565 THAs were followed up for at least one month. The demographic and preoperative details of all patients are shown in Table 1.

### 3.2. Operative Time

There was a significant difference in operation time (Table 2) among the groups. The mean operative time was longer (*p* < 0.001) for patients who were morbidity obese than for patients in the other four groups.

### 3.3. Blood Loss

The comparison of blood loss (Table 2) between the different BMI groups also showed a significant increase in the morbidly obese group with the mean of 376 mL (SD 186), with 302 mL (SD 132) in the normal-weight group. There was no significant difference between the groups regarding the length of hospital stay (ANOVA, *p*=0.531).

### 3.4. Complications

We observed an overall complication rate of 2.8% (*n* = 44) in our series. The complications are listed in Table 3. When stratified according to BMI, the complication rate was higher in morbidly obese patients. Overall, 0% of patients in the underweight group, 2.4% of patients in the normal-weight group, 3.1% of overweight patients, and 2.5% of class I obesity patients had complications, compared to 8.9% of morbidly obese patients.

Adjusted odds of overall complications among different BMI groups showed no significant differences after controlling confounding factors. Associations between individual complications and BMI categories were not examined in the multivariate logistic regression model, as individual complications occurred too infrequently. In contrast, longer operative time (OR 1.02, 95% CI 1.006–1.034, *p*=0.006), older age (OR 1.027, 95% CI 1.001–1.054, *p*=0.041), and cardiovascular comorbidities (OR 2.443, 95% CI 1.162–5.132, *p*=0.018) were found to be associated with overall 30-day complications (Table 4).

The incidence of periprosthetic infection was higher in the morbidly obese group than in the other four groups: 0% (*n* = 0) for underweight patients, 0.14% (*n* = 1) for normal-weight patients, 0% (*n* = 0) for overweight patients, and 0% (*n* = 0) for class I obesity patients, compared with 2.2% (*n* = 1) in morbidly obese patients.

The dislocation rate was 0.19% (*n* = 3) in this investigation. Notably, 3 patients experienced dislocations on average 3 weeks following the index surgery and all occurred in the normal-weight patients. Two patients were treated with closed reduction successfully. One patient experienced recurrent dislocation and achieved stability after revision hip arthroplasty. There was no statistically significant difference in the dislocation rate between the groups.

## 4. Discussion

The obesity epidemic is becoming a worldwide phenomenon and has been identified as a national health problem [28, 29]. The prevalence of obesity has been increasing sharply in Taiwan over the past 20 years [30]. Due to the association of obesity with the development of osteoarthritis, the obese population will be at greater risk of total joint arthroplasty and comprise an ever-increasing segment of the total joint arthroplasty population [31]. Moreover, obesity increases the likelihood of a number of diseases, including diabetes, cardiovascular disease, obstructive sleep apnea, and cancer, which adds to the complexity of caring for patients undergoing total joint arthroplasty [32, 33]. On the other hand, underweight patients undergoing THAs are also a challenging problem due to their potential osteoporosis and malnutrition status that may incur additional postoperative complications. Understanding the factors associated with perioperative complications and causes of 30-day readmission after primary THA may enable preemptive strategies to mitigate preventable causes for unplanned visits and readmissions [34–37]. The primary aim of this study was to evaluate whether extreme BMI levels are associated with a high incidence of 30-day readmission or major events in patients undergoing primary THA. To our knowledge, this is the largest reported single-surgeon study of perioperative complications after THA.

Literature regarding the impact of obesity on the occurrence of perioperative complications following THA is abundant. Perioperative complications and readmission rates have been widely reported to be higher in the setting of obese patients undergoing arthroplasty. Some studies have reported an increased risk of postoperative complications among obese patients [38–42]. However, other studies have argued that there are no differences in complication rates [18–20]. A recent meta-analysis of prospective cohort studies reported that obesity negatively influences the overall complication rate, dislocation rate, functional outcome, and operative time of primary THA [14]. Another meta-analysis, including not only prospective studies but also retrospective studies, has reported that obesity appears to have a negative impact on the outcome of total hip replacement procedures [13]. Our study demonstrates that obese patients have a higher risk of perioperative complications after THA (morbidly obese group 8.9% vs. normal-weight group 2.4%). However, there is no significant difference in odds for 30-day complication between those who are morbidly obese and normal-weight patients (Table 4).

There was a trend toward a higher incidence of acute periprosthetic infection in morbidly obese patients (2.2%) in our study although these patients represented only 2.9% of those undergoing primary THA. A high rate of infection was previously reported in a meta-analysis evaluating THA in obese patients with a BMI of above 30 [13, 14]. Obesity has been reported to be an independent risk factor for periprosthetic infection and all-cause readmission after primary hip arthroplasty [36, 41]. The increased technical difficulty of THA in obese patients requiring longer operative times has also been reported [43, 44]. The findings of our study showed that the operative time was longer in morbidly obese patients than that in other groups (*p* < 0.001). In addition, increased operative time has been reported to be an independent risk factor for periprosthetic joint infection [44–46] and venous thromboembolism [47] after total joint arthroplasty and remains a potentially modifiable risk factor of interest to orthopedic surgeons. A recent study investigated the effects of operative time on short-term complications after total joint arthroplasty [44]. Operative time >120 minutes was associated with increased short-term morbidity and mortality after primary total joint arthroplasty. Moreover, operative time correlated with increased intraoperative blood loss [48, 49]. Hrnack et al. [48] retrospectively analyzed 78 primary THA patients subjected to a mean operative time of 134 minutes and found that increases in operative time were associated with greater intraoperative blood loss. Of particular concern to orthopedic surgeons is the association of obesity with dislocation after primary THAs. Our findings with regard to dislocations following THAs in obese patients differ from previous studies [13, 14]. There were no significant differences in dislocation rates among different BMI groups. Furthermore, higher surgeon volume was significantly associated with a lower rate of dislocation and deep hip infection [50].

Whether underweight (BMI <18.5) patients undergoing primary THAs are associated with an increased risk of complications remains the issue of much scrutiny. A low BMI may be the reflection of a state of malnourishment where the body has fewer reserves, cannot react to stress appropriately, and may have a weakened immune system [51]. Musculoskeletal consequences of being underweight include less muscle mass, less soft tissue, and greater probability of osteoporosis [51, 52]. Anoushiravani et al. in a study comparing complications after THAs among underweight and normal BMI patients, demonstrated that underweight patients were more likely to have postoperative cardiac complications [22]. A study by Alfonso et al. demonstrated that a BMI <18.5 kg/m^2^ was associated with significantly increased posterior dislocation and medical complications [21]. In our study, patients with a BMI <18.5 undergoing primary THA did not present any complications. Comparison of operation time, blood loss, length of stay, and complications of the underweight group and the normal-weight group showed no difference. As there was a relatively smaller percentage (3.6%, *n* = 1565) of the underweight population in our study sample, higher patient numbers are necessary to achieve adequate statistical power to verify this conclusion.

As patients age, the prevalence and severity of comorbid illness are increasing, leading to perioperative complications. In a national database analysis, Boniello et al. [53] compared the rate of 30-day complications and mortality between patients over 80 years of age and under 80 years of age who underwent total hip arthroplasty. They found that “age over 80 years were 1.41 times more likely to sustain complications and 2.02 times more likely to die” than the younger cohort and cardiac comorbidities were independent risk factor for readmission within 30 days. Our result is in accordance with the above database analysis, indicating that advanced age and cardiovascular comorbidities are independent risk factors for perioperative complications after THA.

BMI is one of the most commonly used surrogate markers for evaluating outcomes following surgery, and obesity is an often-cited cause of surgical morbidity [54–57]. Notably, patients with obesity often have an increased incidence of multiple comorbidities including type 2 diabetes, cancer, and cardiovascular diseases [58]. As a result, the utility of BMI alone as a predictor of unfavorable outcomes following THA is under scrutiny. Our study identified more meaningful risk factors that predict unfavorable early outcomes following THA, including advanced age and cardiovascular comorbidities. Based on our findings, we suggest that clinicians avoid using BMI alone for risk assessment for patients undergoing THAs. Instead, our data indicate that advanced age and cardiovascular comorbidities can predict perioperative outcome more accurately.

A potential weakness of this investigation was the fact that complications or readmissions beyond the 30 days after the index surgery were not recorded. Extreme BMI groups, underweight and morbidly obese patients, represent only a small proportion of patients undergoing THA, with only 56 (3.6%) and 45 (2.9%) in the underweight patients and morbidly obese patients, respectively. The number of individual complications was not sufficiently large to perform multivariable analysis and identify specific risk factors associated with complications with adequate statistical power. ASA classification has been criticized for their subjective nature [59]. Although this is a single-surgeon series, there were more than 10 anesthesiologists involved in the care of the patients and the criteria for ASA scoring might not be standardized among them. As such, matching patients based on ASA score is less appropriate in our study. Despite these limitations, our study describes the largest single-surgeon cohort of patients undergoing primary THA, eliminating limitations generally encountered by large administrative databases, such as coding accuracy, access to detailed medical records, and different levels of surgical experience.

## 5. Conclusion

We found that patients with BMI ≥35.00 kg/m^2^ undergoing THA are associated with longer operative time and more blood loss during procedures, but not with longer hospital stay. The most important finding in our series is that BMI alone does not affect 30-day complications after THA. Moreover, our study does not provide evidence of higher rates of 30-day complications in underweight patients. Operation time, blood loss, and length of hospital stay are comparable to normal-weight patients. Advanced age, prolonged operative time, and cardiovascular comorbidities are at increased risk of complications. We should inform patients with above information when undergoing THA. Withholding THA simply on the basis of BMI is not justified.

## Figures and Tables

**Table 1:** Demographics, preoperative comorbidity, and operative variables in different BMI groups.

BMI	<18.5	18.5–24.99	25–29.99	30–34.99	>35	*p* value
Number of patients	56	697	609	158	45	

Mean BMI (SD), kg/m^2^	17.35 (1.28)	22.57 (1.66)	27.21 (1.41)	31.95 (1.29)	37.60 (2.94)	<0.001^a^

Mean age (SD, range), y	47.0 (16.8, 22–83)	54.6 (14.6, 16–91)	57.5 (13.2, 15–87)	56.0 (14.0, 21–87)	57.2 (12.2, 26–77)	<0.001^a^

Sex, *n* (%)						<0.001^a^
Male	23 (41.1)	289 (41.5)	327 (53.7)	80 (50.6)	16 (35.6)	
Female	33 (58.9)	408 (58.5)	282 (46.3)	78 (49.4)	29 (64.4)	

Pathology, *n* (%)						
OA	3 (5.4)	110 (15.8)	135 (22.2)	24 (15.2)	7 (15.6)	
Osteonecrosis	35 (62.5)	317 (45.5)	215 (35.3)	66 (41.8)	11 (24.5)	
Inflammatory	11 (19.6)	34 (4.9)	32 (5.3)	6 (3.8)	2 (4.4)	
Dysplasia	7 (12.5)	212 (30.4)	192 (31.5)	57 (36.1)	24 (53.3)	
Perthes disease	0	10 (1.4)	13 (2.1)	2 (1.3)	0	
FAI	0	9 (1.3)	17 (2.8)	3 (1.8)	1 (2.2)	
OA hip secondary to childhood septic hip	0	5 (0.7)	5 (0.8)	0	0	

Fixation, *n* (%)						0.58
Cemented	0	1 (0.1)	0	0	0	
Hybrid	0	8 (1.2)	2 (0.3)	1 (0.6)	0	
Cementless	56 (100)	688 (98.7)	607 (99.7)	157 (99.4)	45 (100)	

ASA score						
1-2	45 (80.4)	536 (77)	498 (81.8)	119 (75.3)	31 (68.9)	0.07
3-4	11 (19.6)	161 (23)	111 (18.2)	39 (24.7)	14 (31.1)	

Comorbidity, *n* (%)						
Cardiovascular comorbidities	11 (19.6)	252 (36.2)	306 (50.2)	103 (65.2)	33 (73.3)	<0.001^a^
Respiratory comorbidities	10 (17.9)	104 (14.9)	82 (13.5)	26 (16.5)	9 (20.0)	0.629
Diabetes	3 (5.4)	71 (10.2)	91 (14.9)	37 (23.4)	17 (37.8)	<0.001^a^

OA, osteoarthritis; FAI, femoroacetabular impingement; SD, standard deviation. ^a^Statistically significant difference (*p* < 0.05).

**Table 2:** Perioperative parameters.

BMI	<18.5	18.50–24.99	25–29.99	30–34.99	>35.00	*p* value	Post hoc
Number of patients	56	697	609	158	45		
Mean operative time (SD, range), min	74.39 (18.54, 44–118)	69.82 (17.59, 30–162)	68.56 (16.35, 42–160)	73.02 (18.59, 40–165)	78.96 (23.68, 47–163)	<0.001^a^	B vs. E
							C vs. E
Mean blood loss (SD), mL	261 (148)	302 (132)	312 (152)	320 (131)	376 (186)	0.001^a^	A vs. E
							B vs. E
Mean hospital stay (SD), *d*	3.54 (0.60)	3.58 (0.63)	3.6 (0.70)	3.52 (0.65)	3.69 (0.67)	0.531	
Number of 30-day complications (%)	0	17 (2.4)	19 (3.1)	4 (2.5)	4 (8.9)	0.129	

^a^Statistically significant difference (*p* < 0.05), A: BMI  < 18.5; B: BMI 18.5‐24.99; C: BMI 25‐29.99; D: BMI 30‐34.99; E: BMI  > 35.

**Table 3:** Summary of 30-day complications.

BMI	<18.5 (*n* = 56)	18.5–24.99 (*n* = 697)	25–29.99 (*n* = 609)	30–34.99 (*n* = 158)	>35 (*n* = 45)
Surgery-related (%)	0	10 (58.8)	8 (42.1)	2 (50)	3 (75)

Surgery-unrelated (%)	0	7 (42.2)	11 (57.9)	2 (50)	1 (25)

Complications or readmission related to surgery					
Intraoperation fracture	0	4	3	2	1
Sciatic nerve injury	0	1	0	0	0
Superficial wound infection	0	1	3	0	0
Deep infection	0	1	0	0	1
Dislocation	0	3	0	0	0
Stem early subsidence	0	0	2	0	1

Complications or readmission unrelated to surgery					
Fractures due to postoperative fall	0	2	3	0	0
Other medical complications	0	5^a^	8^b^	2^c^	1^d^

^a^1 acute kidney injury, 1 cholecystitis, 1 gastrointestinal bleeding, 1 vertigo, 1 renal insufficiency. ^b^2 acute myocardial infarction, 3 pneumonia, 1 gastrointestinal bleeding, 1 vertigo, 1 depression. ^c^1 stroke, 1 pneumonia. ^d^1 acute myocardial infarction.

**Table 4:** Multivariate logistic regression analysis identifying independent risk factors for complications within 30 days.

Variable	Odds ratio (95% CI)^a^	*p* value
BMI		
18–24.99	Reference	
<18.5	—	—
25–29.99	1.12 (0.571–2.197)	0.742
30–34.99	0.762 (0.247–2.346)	0.635
>35	2.415 (0.742–7.862)	0.143
Age	1.027 (1.001–1.054)	0.041
Cardiovascular comorbidities	2.443 (1.162–5.132)	0.018
Operative time	1.02 (1.006–1.034)	0.006

^a^CI, confidence interval.

## Data Availability

The data used to support the findings of this study are available from the corresponding author upon request.
